# Effect of Prebiotic Supplementation With and Without Physiotherapy on Pain and Pain Sensitivity in People with Knee Osteoarthritis

**DOI:** 10.3390/nu18050714

**Published:** 2026-02-24

**Authors:** Afroditi Kouraki, Susan Franks, Amrita Vijay, Thomas Kurien, Moira A. Taylor, Stephanie L. Smith, Benjamin Smith, Anthony Kelly, Ana M. Valdes

**Affiliations:** 1 NIHR Nottingham Biomedical Research Centre, School of Medicine, University of Nottingham, Nottingham NG7 2UH, UK; susan.franks@nottingham.ac.uk (S.F.); amrita.vijay@nottingham.ac.uk (A.V.); thomas.kurien@nottingham.ac.uk (T.K.); moira.taylor@nottingham.ac.uk (M.A.T.); stephanie.smith2@nottingham.ac.uk (S.L.S.); benjamin.smith@nottingham.ac.uk (B.S.); tony.kelly@nottingham.ac.uk (A.K.);; 2 Academic Unit of Injury, Recovery and Inflammation Sciences, School of Medicine, University of Nottingham, Nottingham NG7 2UH, UK; 3 Arthritis UK Pain Centre, University of Nottingham, Nottingham NG5 1PB, UK; 4 Division of Trauma and Orthopaedics, Nottingham University Hospitals NHS Trust, Nottingham NG5 1PB, UK; 5 The David Greenfield Human Physiology Unit, School of Life Sciences, University of Nottingham, Nottingham NG7 2TQ, UK; 6 University Hospitals of Derby and Burton NHS Foundation Trust, Derby DE22 3NE, UK

**Keywords:** knee osteoarthritis, 2 × 2 factorial design, physiotherapy-supported exercise, prebiotic supplements, pain, inulin, fibre, glucagon-like peptide-1, short-chain fatty acids

## Abstract

**Background**: Emerging evidence links the gut microbiome to chronic pain processing. Inulin, a prebiotic fibre, modulates the gut microbiome, while physiotherapy-supported exercise (PSE) improves pain and function. We evaluated the effects of inulin supplementation with and without PSE on knee osteoarthritis (OA) pain. **Methods**: In a 2 × 2 factorial RCT, 117 community-dwelling adults with knee OA received 6 weeks of: (A) 20 g/day inulin, (B) digital PSE (Joint Academy™), (C) inulin +PSE, or (D) 10 g/day maltodextrin. Primary outcome: pain (Numerical Rating Scale). Secondary: 30 s sit-to-stand (30-CST), timed up and go (TUG), grip strength, and quantitative sensory testing. Serum short-chain fatty acids (SCFAs) and glucagon-like peptide-1 (GLP-1) were measured. The study was not powered to detect synergistic interaction. **Results**: A total of 117 participants (58.1% female; mean ± SD age = 67.5 ± 9.4 years; BMI = 29.5 ± 5.3 kg/m^2^; NRS = 3.96 ± 2.67) completed the trial. Pain improved with inulin (baseline-adjusted between-group mean difference (Δ) = −1.11 [95%CI −2.18, −0.04], *p* = 0.045) and PSE (Δ = −1.55 [95%CI −2.52, −0.58], *p* = 0.002) compared to placebo, with no synergistic effect. PSE improved TUG (*p* = 0.02) and 30-CST (*p* = 0.0004), while inulin improved grip strength (*p* = 0.002), pressure pain thresholds (*p* = 0.009) and temporal summation (*p* = 0.025) compared to placebo and had significantly lower dropout rates (3.6%) compared with PSE (21% *p* < 0.01). Only inulin increased SCFA butyrate (*p* = 0.0248) and GLP-1 (*p* = 0.0109), and higher GLP-1 was associated with improved grip strength, suggesting a gut–muscle link. **Conclusions**: Inulin and PSE each produced meaningful pain reductions. Only inulin improved pain sensitivity and grip strength, the latter paralleled by increased GLP-1, and had much higher rates of retention compared to PSE.

## 1. Introduction

Knee OA is one of the most prevalent conditions characterised by joint degeneration, leading to chronic pain, stiffness, and reduced mobility. It significantly reduces quality of life and imposes a substantial personal and socioeconomic burden [1]. Non-pharmacological interventions are the first line of treatment [1], and exercise has long been recognised for its benefits in strengthening muscles, improving joint function, and reducing pain [2]. There is extensive evidence for the effectiveness of exercise for the management of knee OA pain [3], whereas dietary approaches have been studied less extensively, and the findings are more mixed. For example, a recent systematic review and meta-analysis reported that while certain supplements such as vitamin D, curcumin, and ginger showed benefits for knee OA pain and function, others like omega-3 and vitamin E did not demonstrate significant effects [4]. This highlights the need to further explore nutritional strategies that may complement established non-pharmacological treatments.

Although few studies exist to date investigating the modulation of the gut microbiome to reduce OA pain, recent research suggests that gut health, influenced by prebiotics, may also play a role in systemic inflammation, pain sensitivity and perception [5,6,7,8]. Several studies have indicated that the gut microbiome can modulate pain and possibly pain sensitivity in animal models [5,6,7,8]. The gut microbiome, a complex community of microorganisms in the digestive tract, plays a crucial role in regulating immune function, inflammation, and neurotransmitter production [9]. An imbalance in gut bacteria (dysbiosis) may contribute to pain and pain sensitisation through its effects on the gut–brain axis [10], with microbial metabolites such as short-chain fatty acids (SCFAs) influencing neural pathways involved in pain perception [11].

Inulin is a soluble prebiotic fibre naturally found in chicory root, Jerusalem artichoke, and other plant sources. It selectively promotes beneficial gut bacteria growth and enhances production of bioactive metabolites, including SCFAs [12].

In addition to these gut microbiome-related effects, glucagon-like peptide-1 (GLP-1), a peptide hormone produced by intestinal L-cells, has emerged as an important signalling molecule of the gut–brain and gut–muscle axes. Dietary fibres, particularly prebiotic types such as inulin, have been shown to enhance GLP-1 secretion in both preclinical and human studies [13,14]. In addition, preclinical evidence indicates that GLP-1, modulated by gut microbiota [15], may regulate nociception [16] and mitigate cartilage degradation [17].

A few interventional clinical studies suggest that prebiotic supplementation may indeed help relieve chronic pain. For example, prebiotic supplementation has been reported to significantly improve pain scores in patients with fibromyalgia [18]. A recent study reported a between-group mean difference in knee pain of −1.1 points at 6 months, favouring prebiotic supplementation (*p* = 0.059) over placebo in adults with knee OA and comorbid obesity [19].

Prebiotics may therefore influence inflammatory and pain modulation pathways, complementing the physical benefits of exercise. We and others have shown that digital delivery of physiotherapy exercise is a safe and effective way of delivering standardised exercises to reduce pain and improve function in individuals with knee OA [20]. However, we found that digitally delivered physiotherapy-supported exercise (PSE) had no effect on pain sensitivity despite a moderate to strong effect in reducing pain. Given that gut microbiome composition could potentially modulate sensitivity, we hypothesised that a prebiotic intervention known to increase levels of SCFAs could also improve pain sensitivity (potentially indicating reduced central sensitisation) in people with knee OA pain.

The main aim of this study was to evaluate whether prebiotic nutritional supplementation, working through distinct gut microbiome mechanisms, could enhance pain outcomes independently or synergistically with PSE. The primary objective was to evaluate the magnitude of the effect of prebiotic supplementation (inulin) side-by-side with PSE versus placebo (maltodextrin) on knee pain. Secondary objectives included exploring potential interaction (synergy) between the two interventions to assess the combined role of prebiotics and physiotherapy, allowing a structured examination of the effects of two independent interventions simultaneously. Additional outcomes included pain sensitisation indices and functional outcomes including walking and squatting ability as well as grip strength. Serum SCFAs and GLP-1 were investigated as potential mechanistic markers of the prebiotic effects.

## 2. Materials and Methods

**Figure 1:**
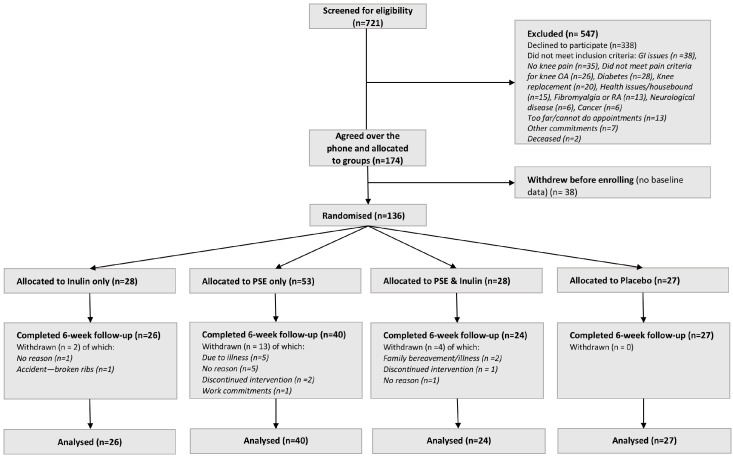
INSPIRE study CONSORT flow diagram. Of 721 participants screened, 547 were excluded prior to randomisation (338 declined to participate and 209 did not meet inclusion criteria). A total of 174 individuals agreed to participate over the phone and were provisionally allocated into groups. Of these, 38 withdrew before enrolment and did not attend the first visit, providing no baseline data. The remaining 136 participants were randomised across four treatment arms. Of these, 117 completed both baseline and follow-up assessments (included in the per-protocol analysis), and 19 withdrew after baseline but before follow-up (included in the intention-to-treat analysis analysis using multiple imputation), corresponding to a dropout rate of 14%. Dropout rates were higher in the physiotherapy arms (17/81; 21%) than in the non-physiotherapy arms (2/55; 3.6%), representing a statistically significant difference (χ^2^(1) = 7.2, *p* = 0.008; Fisher’s exact test *p* = 0.008).

The study was performed according to the Declaration of Helsinki and received approval from the University of Nottingham Faculty of Medicine & Health Sciences Research Ethics Committee (REC) (Ref: 473-0322). The study was registered with ClinicalTrials.gov reference number NCT05670314. All participants provided written informed consent. Clinical visits were conducted at the Clinical Sciences Building, City Hospital, Nottingham, UK. This study followed the Consolidated Standards of Reporting Trials (CONSORT) reporting guideline (Figure 1).

### 2.1. Participants

Participants were recruited either from pre-existing institutional databases of community-dwelling individuals who had given consent to be contacted for research on chronic pain or through responses to advertisements posted on social media.

### 2.2. Trial Design

The INSPIRE study is a 2 × 2 factorial RCT in the community setting at Nottingham with participants identified as having knee pain from OA. Participants were initially randomised in a 1:1:1:1 ratio to web-based PSE, inulin, PSE and inulin or placebo and usual care. During the course of the trial, substantially higher dropout was observed in the PSE arms (see Supplementary Materials). To achieve the prespecified target sample size and maintain statistical power, allocation probabilities were modified during later recruitment waves to preferentially recruit participants into the PSE arms. Details of the study are presented in the protocol published in ClinicalTrials.gov (NCT05670314) [21].

### 2.3. Inclusion and Exclusion Criteria

Participants were provisionally allocated to groups following a telephone screening confirming they met the study’s inclusion and exclusion criteria. Formal randomisation occurred at the baseline visit, where eligibility was re-confirmed in person (see Supplementary Table S1 for detailed inclusion and exclusion criteria). Recruitment started in May 2022 and enrolment ended in February 2025. The trial ended when the required sample size was reached. After 80% of participants had completed both study visits, it became apparent that dropout rates were over five-fold higher in the PSE arms compared to the non-PSE arms. To achieve the target sample size and maintain adequate statistical power, it was necessary to extend recruitment specifically for the PSE arms (see also Supplementary Materials).

### 2.4. Interventions

Prebiotic intervention: Inulin fibre (20 g) in powder form (commonly found in root vegetables such as chicory) was randomly allocated to eligible participants. They were instructed to mix it into breakfast cereal, smoothies, yogurt, or a drink of their choice. This dosage is standard and has been shown to effectively modulate the gut microbiome and metabolic markers in previous studies of inulin supplementation in adults [22].

PSE intervention: The study used a digitally delivered physiotherapy platform known as Joint Academy (JA) [https://www.jointacademy.com/gb/en/ (accessed on 18 February 2026)] as a previous RCT from our group, which demonstrated promising results with this platform [20]. The JA company granted permission for this study to be carried out using their platform. The standardised PSE intervention, previously described in detail [20], includes tailored intensity levels and combines concentric and eccentric exercises with open- and closed-chain movements to strengthen the legs, including the hip and knee muscles, and improve balance. The programme also includes educational sessions on OA fundamentals, treatment, symptom self-management, and healthy lifestyle benefits. Participants received an email with a link to the online platform and log-in instructions. The PSE intervention, lasting 6 weeks, began after log-in and a kick-off call with a personal physiotherapist, and participants were expected to engage daily.

Placebo group: Participants in the control placebo group continued with their usual self-management (community setting) and received maltodextrin (10 g) daily in powder form (a low-digestible glucose polymer made from starches commonly found in corn, potatoes and rice), with similar consumption instructions as inulin. Due to potential postprandial glycaemic effects of maltodextrin [23], a lower dose of 10 g was selected for ethical reasons.

At the first visit, both groups received pre-measured weekly pots containing either supplement or placebo, along with scoops. Participants were instructed to take 10 g/day of maltodextrin or 20 g/day of inulin daily for 6 weeks, depending on their assigned group.

Intervention adherence: Adherence to the PSE was monitored via the JA app, recording the number of days participants actively engaged in treatment, while adherence to inulin and maltodextrin was monitored through dietary compliance records. Participants were requested to consume their usual dietary intake during the period of the intervention.

### 2.5. Outcomes

Primary outcome: The Numerical Rating Scale (NRS) for pain was used as the primary outcome. It was assessed using the question “On average, how much pain have you experienced in your most painful knee over the past week? Please indicate your pain level on the following scale”. Participants reported their pain on a scale ranging from 0 to 10, where 0 represents no pain and 10 indicates the worst pain imaginable. This measure was used at both visits to evaluate changes in pain levels in response to the interventions from baseline to 6 weeks.

Secondary outcomes assessed during the baseline and follow-up visits as described below included measures of quantitative sensory testing (QST) and changes in functional outcomes (30 s sit-to-stand test (30-CST), timed up and go (TUG) test, grip strength).

QST is a non-invasive method to assess pain sensitivity using standardised stimuli like mechanical pressure or sharpness. QST modalities used were the pressure pain detection threshold (PPT) at five anatomical sites (proximal and distal to tissue pathology) and temporal summation (TS) as previously described [24]. Both tests demonstrate high to very high reliability in measuring pain sensitivity across various testing sites and musculoskeletal conditions [24].

The PPT measures the lowest pressure a participant perceives as painful while pressure is applied using a handheld probe (Medoc-AlgoMed, Ramat Yishai, Israel) at a rate of 50 kPa/s on the most painful knee, with lower PPTs suggestive of increased pain sensitivity. TS assesses sensitivity to sharpness by applying a brief “pinprick” stimulus (256 mN Pinprick; MRC-Systems, Heidelberg, Germany) to the skin, with higher ratings potentially suggesting increased spinal cord pain sensitivity (for more details see Supplementary Materials).

30-CST and TUG: The 30-CST measures how many times a participant can rise from a chair to a full standing position within 30 s. At enrolment, participants received a demonstration and practiced once before completing a single trial to prevent fatigue. The TUG test assesses the time (seconds) taken for a participant to stand up on command, walk 3 m, turn, return to the chair, and sit down. The test was performed three times, with the average time recorded. Both 30-CST and TUG are validated methods for assessing lower body strength and functional mobility [25,26].

Grip strength: Hand-grip strength was measured using a hand dynamometer (Lafayette Instrument^®^, Model J00105, Lafayette, IN, USA). Participants sat upright in a stable chair with thighs horizontal and knees at 90 degrees and the non-tested arm resting in their lap. The tested arm was positioned with the upper arm vertical, lower arm horizontal, and elbow tucked into the waist. They were instructed to squeeze as hard as possible momentarily and then release. This was repeated three times on the dominant hand, with the average recorded. This method has been shown to have high reliability [27].

Knee injury and Osteoarthritis Outcome Score (KOOS): The KOOS is a self-administered questionnaire designed to assess patient-reported outcomes in individuals with knee injury and osteoarthritis. It consists of 42 items divided into five subscales: Pain, Other Symptoms, Activities of Daily Living, Sport and Recreation Function, and Knee-related Quality of Life. Each item is scored on a 0–4 Likert scale and transformed to a 0–100 score, with lower values indicating worse pain or function. The KOOS has been extensively validated for knee OA populations, showing excellent internal consistency (Cronbach’s α > 0.8), test–retest reliability (ICC > 0.85), and responsiveness to both pharmacological and non-pharmacological interventions, and is widely used in clinical and epidemiological studies [28]. In this study, the KOOS was administered at baseline and six weeks to assess self-perceived changes in pain and functional status. The KOOS was collected as an exploratory patient-reported outcome and analysed descriptively. The KOOS was not a prespecified primary or secondary outcome in the trial registry and was therefore not used to support efficacy conclusions.

SCFAs: Fasting blood samples were collected at baseline and follow-up visits by trained staff using 6 mL Gold Vacutainer serum separation tubes. After resting for 30 min, the samples were centrifuged to obtain serum aliquots and stored in −80 °C freezers following local standard operating procedures. Targeted SCFA profiling was conducted on serum samples at the Edinburgh Mass Spec Core Facility, University of Edinburgh. The analysis utilised liquid chromatography coupled with high-resolution mass spectrometry, following the protocol detailed by Billen et al. [29]. After derivatisation with 3-nitrophenylhydrazine, the SCFAs quantified included acetate, propionate and butyrate.

GLP-1: Following the observation that serum short-chain fatty acids did not fully explain the pain-relieving effects of inulin supplementation, we conducted an exploratory analysis of glucagon-like peptide-1 (GLP-1) as a potential mechanistic biomarker. As this was not prespecified in the original protocol, GLP-1 findings should be considered hypothesis-generating and require validation in future studies.

Serum aliquots were analysed for GLP-1 levels using a sandwich ELISA performed by Affinity Biomarkers. The Millipore Human GLP-1 Total Immunoassay (catalogue no. EZGLP1T-36; EMD Merck-Millipore Corporation, Burlington, MA, USA) was used to capture GLP-1 molecules with an anti-GLP-1 polyclonal antibody, followed by detection with a biotinylated antibody and horseradish peroxidase conjugation. Enzyme activity was measured spectrophotometrically, and GLP-1 concentrations were quantified by comparison to a standard reference curve. Fasting GLP-1 concentrations typically range from 0 to 15 pmol/L. The assay demonstrates high intra- and inter-assay precision, as assessed using samples of known concentration. The quantification range extends up to 1000 pmol/L, with values below 1.5 pmol/L reported as <1.50 pmol/L. The assay specifically measures GLP-1 (7–36) and GLP-1 (9–36) amide forms, with minimal cross-reactivity to related peptides.

### 2.6. Sample Size

The a priori power calculation for the 2 × 2 factorial design with 30 individuals per block (total *n* = 120), assuming ≤10% dropout, has 80% power to detect differences of 0.51 standard deviations (SDs) with alpha < 0.05. This effect size was deemed reasonable compared to the observed effect size in our previous digital PSE intervention (a drop of 0.84 SDs in the PSE arm compared with only 0.2 SDs in the control arm who only received a leaflet). In addition, the study was adequately powered to detect moderate effects of inulin on pain outcomes, with 30 participants per group providing ~75–80% power to detect an effect size of Cohen’s d~0.6 at α = 0.05.

### 2.7. Randomisation and Blinding

Randomisation was performed using simple computer-generated randomisation without restrictions or stratification and allocation concealment software called “sealed envelope” [https://www.sealedenvelope.com/ (accessed on 18 February 2026)]. Generation of a randomisation schedule included obtaining the random numbers and assigning them to each subject under the specific conditions. Different members of the research team were responsible for sequence generation, participant enrolment, and intervention assignment, separate from those conducting outcome assessments. Researchers assessing outcomes were blinded to the interventions and participants were blinded to the dietary intervention. However, blinding for the PSE intervention was not feasible for participants since they were informed if they were assigned to one of the PSE groups or not. Participants who were randomly assigned to the non-PSE arms were offered access to the web-based PSE programme after completing the second study visit. Thus, outcome assessors remained blinded to both interventions, whereas participants were blinded only to inulin versus placebo allocation.

### 2.8. Patient and Public Involvement Statement

The study design was informed by 18 qualitative interviews with iBEAT-OA trial participants who completed PSE [20]. The interviews revealed a strong interest in using nutritional strategies to manage knee OA pain, prompting the inclusion of inulin in the current study. Two patient collaborators contributed to drafting the initial protocol and reviewed the final participant-facing documents with all their feedback fully incorporated.

### 2.9. Additional Measures for Future Analyses

Additional protocol-specified measures were collected for a smaller subset of participants, including changes in gut microbiome diversity, serum endocannabinoid profiles, serum inflammatory protein levels, and expression levels of predefined pain-related genes (RNA sequencing of blood samples). Absolute changes for these measures are available in the protocol results for all groups (ClinicalTrials.gov NCT05670314). These measures did not demonstrate significant changes or associations with the primary outcome reported here and will be included in a planned secondary analysis examining moderators and predictors of treatment response. In addition, although the Hospital Anxiety and Depression Scale (HADS) was included in the protocol as a supporting psychosocial assessment, it was not listed as a primary or secondary outcome in the trial registry and was not analysed as an efficacy endpoint. Accordingly, HADS results are not reported in this manuscript.

### 2.10. Statistical Analysis

The primary outcome was pain assessed on a 0 to 10 NRS at 0 and 6 weeks. An analysis of covariance (ANCOVA) was used to assess differences in follow-up outcome measures among treatment groups while adjusting for baseline levels [as per the analysis plan included at the time of protocol submission; https://clinicaltrials.gov/study/NCT05670314?term=NCT05670314&rank=1#more-information (accessed on 18 February 2026]. The model included treatment group (arm: inulin only, PSE only, inulin and PSE, control/placebo) as a categorical independent variable and baseline outcome scores as a covariate. Model assumptions were evaluated through residual diagnostics to ensure the validity of the analysis. Individuals missing data on the primary outcome at either baseline or follow-up were excluded from the per-protocol analysis. Secondary outcomes were analysed in the same way, and in addition, as the study was not powered to detect these outcomes, simple comparisons between inulin and no-inulin or PSE and no-PSE were performed using standard parametric (*t*-tests) or non-parametric (Mann–Whitney) methods where change from baseline to follow-up was not normally distributed. Although recruitment started in 2022, the protocol and statistical analysis plan were refined and updated after a 2023 ethics amendment for clarity, including additional post-intervention follow-up questionnaires while maintaining the prespecified analytical approach.

Responder sub-analysis: In addition to the continuous analysis of NRS pain scores, we conducted a responder sub-analysis to assess the proportion of participants achieving a pain reduction. We defined a decrease of ≥2 points on the NRS from baseline to follow-up as “response”. This very stringent threshold was selected based on the placebo group response pattern, where 33% of placebo participants (9/27) (vs. ~0% in the treatment arms) showed a reduction of ≥1 point but only 11% achieved a reduction of ≥2 points (3/27), suggesting that the higher threshold is more likely to reflect true physiological changes in pain rather than placebo effects or measurement variability. Between-group differences in responder rates were analysed using chi-squared tests, with results presented as proportions and corresponding *p*-values for comparisons with placebo.

Software used: R studio version 4.4.1 and Graph Pad PRISM v10 were used for the statistical analyses. No generative AI was used in the preparation of this manuscript.

## 3. Results

Among 117 participants randomly assigned to groups, the mean (SD) age was 67.5 (9.4) years and BMI was 29.5 kg/m^2^ (5.3) at baseline. Slightly more than half of the participants were female (58.12%) and the average pain score at baseline was an NRS mean (SD) of 3.96 (2.67). The descriptive characteristics of each of the study arms are shown in Table 1. There were no significant differences in age, sex, BMI or Index of Multiple Deprivation (IMD) between the arms. Pain sensitisation indices (TS and PPTs) and the KOOS function are shown in Supplementary Table S3 (Supplementary Materials). There were no significant differences in baseline functional assessments (TUG, 30-CST, grip strength, KOOS) or pain traits (NRS, TS and PPTs) (Table 1 and Table S3).

Of 721 participants screened, 547 were excluded. At 6 weeks, data from all randomised participants were assessed in statistical analysis. Among the 136 participants who were randomised, 117 completed both baseline and follow-up assessments, while 19 withdrew after baseline but before follow-up, corresponding to an overall dropout rate of 14%. To account for missing data and assess the robustness of the complete-case (per-protocol) analysis, a sensitivity intention-to-treat (ITT) analysis was conducted including all 136 randomised participants, with missing outcomes for the 19 withdrawn participants imputed using multiple imputation. Results were consistent in direction and magnitude with the per-protocol findings (see Supplementary Table S2 and Figures S1 and S2, Supplementary Materials).

The participant flowchart with reasons for exclusion is presented in Figure 1 (details on randomisation and dropout rates are described in the Supplementary Materials). There were no differences in main baseline characteristics (age, sex, BMI and NRS pain) between the participants who completed the study compared with those that withdrew or were excluded from the per-protocol analysis (Supplementary Table S4, Supplementary Materials). The enrolment of study completers per arm over time (months 1 to 6, months 7 to 12 and month 13 and beyond) is shown in Supplementary Table S5. There was no significant difference in the descriptive characteristics of participants when stratified by phase of recruitment (Supplementary Table S6).

The mean adherence (i.e., active engagement with the intervention for those who remained enrolled) to the PSE arm was 84% (SD = 18%), while mean adherence to the inulin group was 88% (SD = 23%). Out of the 50 participants that completed the inulin intervention, 23 participants reported very minor gastrointestinal adverse events related to the intervention (e.g., wind and bloating) which did not lead to non-compliance or withdrawal, while 2 reported positive effects (pain-free at night and more regular and comfortable bowel movements). No adverse events were reported for all other non-inulin groups. There were no differences in habitual dietary intake, as assessed by food diaries before and after the intervention, nor were there any differences in the use of pain medications (including opioids, NSAIDs, and anti-neuropathic drugs) among the four groups (see Supplementary Materials).

### 3.1. Primary Outcome

**Figure 2:**
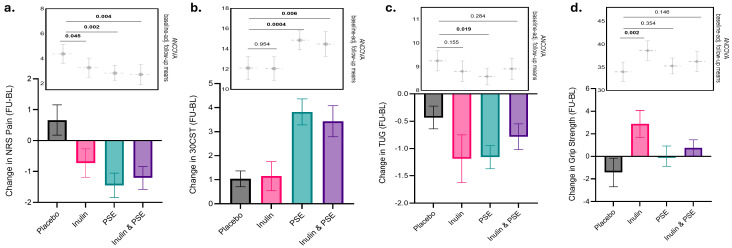
Changes in NRS pain and functional outcomes across treatment groups: Barplots illustrating the changes from baseline to follow-up of (**a**) NRS Pain scores, (**b**) the 30-s sit-to-stand (30-CST) test, (**c**) the timed-up-and-go (TUG) test, and (**d**) grip strength, with comparisons of adjusted means from ANCOVA shown as grey dots indicating the mean at follow-up accounting for baseline scores, with error bars representing the 95% confidence intervals (CI). *p*-values from ANCOVA for all comparisons with the placebo group are shown. Analysis was done by original assigned groups. Abbreviations: PSE = physiotherapy-supported exercise; NRS = Numerical Rating Scale; TUG = timed up-and-go; 30-CST = 30-s sit-to-stand.

A reduction in NRS pain scores over time was observed in the inulin intervention group compared to placebo (Δ = −1.11 [95% confidence interval (CI) =−2.18, −0.04], *p* = 0.045), after adjusting for baseline NRS pain scores, corresponding to a moderate effect size (Cohen’s d = 0.57), indicating that inulin is effective in reducing pain. In the PSE group, a moderate to large effect was observed (Δ = −1.55 [95% CI = −2.52, −0.58], *p* = 0.002, Cohen’s d = 0.72) when compared to placebo, after adjusting for baseline NRS pain scores. Both interventions exceeded the 1-unit minimum clinically important difference (MCID) on the NRS, indicating meaningful clinical improvement. PSE approached the “much better” threshold of 2 units, while inulin met the lower end [30]. The combined treatment with inulin and PSE showed a strong effect in reducing pain scores as compared to placebo (Δ = −1.67 [95% CI = −2.77, −0.57], *p* = 0.004, Cohen’s d = 0.84), after adjusting for baseline NRS pain scores (Figure 2a). A response sub-analysis showed similar results, with all treatment arms having significantly higher response rates than placebo (Supplementary Table S8).

When comparing the combined effect of inulin and PSE exercise to the combined effects of inulin and PSE alone we found no additional synergistic effect (Δ = −1.00 [95% CI = [−2.48, 0.48], *p* = 0.18). However, a post hoc power analysis for the synergistic effect found that the study was underpowered to detect a synergistic interaction (see Supplementary Materials). Therefore, the synergistic interaction results should be considered exploratory. The ANCOVA baseline-adjusted follow-up means are shown in Figure 2a, alongside the raw absolute mean changes in NRS pain score for each treatment arm, which are also reported in Supplementary Table S7 (Supplementary Materials). These analyses were robust to time of recruitment (Supplementary Table S10).

### 3.2. Secondary Outcomes

There was an improvement in squatting ability (30-CST) for PSE over time (Δ = 2.76 [95% CI = 1.30, 4.22], *p* = 0.0004) and with the combination of PSE and inulin (Δ = 2.37 [95% CI = 0.71, 4.05], *p* = 0.006) when compared to placebo, both demonstrating large effects (Cohen’s d = 0.96 and 0.96, respectively), adjusting for baseline 30-CST. However, inulin alone showed no effect when compared to placebo, suggesting that PSE is driving the improvement in squatting ability observed in the inulin plus PSE arm (Figure 2b).

We also observed improvements in walking ability, as measured with the TUG test, with PSE over time when compared to placebo (Δ = −0.66 [95% CI = −1.20, −0.12], *p* = 0.0195, Cohen’s d = 0.59), whereas no effects were found for inulin or inulin plus PSE, adjusting for baseline TUG (Figure 2c).

In contrast, inulin resulted in improvements in grip strength over time compared to placebo (Δ = 4.62 [95% CI = 1.67, 7.57], *p* = 0.002), with a moderate effect size (Cohen’s-d = 0.68), whereas neither PSE nor the combination of the two interventions had an effect on grip strength, adjusting for baseline grip strength (Figure 2d).

A sensitivity intention-to-treat (ITT) analysis including all 136 randomised participants (including the 19 dropouts with baseline-only data), with missing values handled by multiple imputation, revealed treatment effects consistent in direction and magnitude with the primary analyses. All results remained statistically significant except for PSE’s effect on TUG, which showed a trend toward significance (*p* = 0.076) in the ITT analysis (see Supplementary Table S2 and Figures S1 and S2, Supplementary Materials). In addition, a sensitivity analysis adjusting for time of recruitment revealed treatment effects consistent in direction and magnitude with the primary analyses, with all previously significant results remaining significant (Supplementary Table S10).

**Figure 3:**
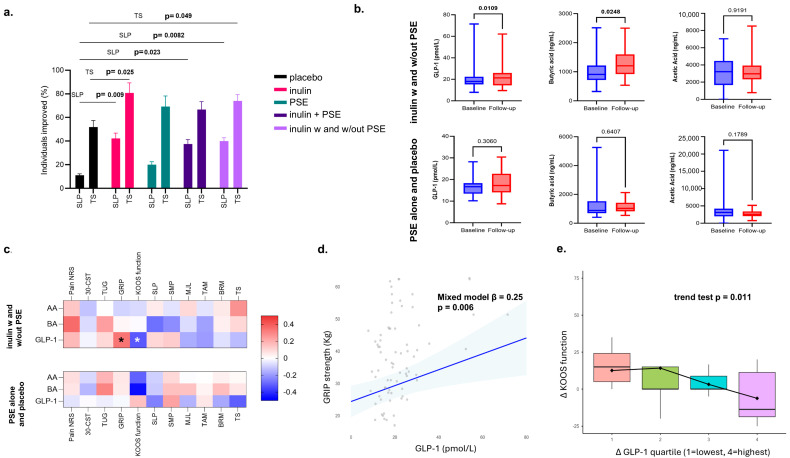
Percentage (%) improvement in pain sensitisation indices, changes in GLP-1 and SCFAs, and associations with pain and function outcomes: (**a**) Percentage of participants who showed improvement in superolateral patella (SLP) pressure pain thresholds and temporal summation (TS) across treatment groups: placebo, inulin, PSE exercise, inulin and PSE, and the combined inulin and inulin & PSE group. *p*-values are derived from chi-squared tests comparing each treatment arm to placebo. (**b**) Boxplots illustrating comparisons of GLP-1 levels (pmol/L) and SCFA levels (ng/mL) from baseline to follow-up in the inulin groups and in the control/PSE groups. Propionic acid not shown as values were below the limit of detection for the majority of individuals. *p*-values are from Wilcoxon tests. (**c**) Heatmap of Spearman’s correlations (Rho) between changes in GLP-1 and SCFA levels with changes in pain and functional outcomes (grip strength, KOOS function, TUG and 30-CST). Nominal associations were observed for GLP-1 with increased grip strength and improved KOOS function; none remained significant after FDR correction. * *p* < 0.05; (**d**) Scatterplot showing the association between higher GLP-1 and improved grip strength in inulin arms based on a mixed-effects model adjusted for age, sex, BMI, group, time, and repeated measures (β = 0.25, *p* = 0.006). The shaded area indicates the 95% confidence interval. (**e**) Boxplot illustrating the relationship between change in GLP-1 and change in the KOOS by quartile of ΔGLP-1 (1 = lowest, 4 = highest), showing a significant trend (*p* = 0.011) in inulin arms, indicating that greater increases in GLP-1 were associated with less difficulty in self-reported knee function. Abbreviations: AA = acetic acid; BA = butyric acid; NRS = Numerical Rating Scale; TUG = timed up and go; 30-CST = 30 s sit-to-stand; GRIP = grip strength; SLP = superolateral patella; SMP = superomedial patella; MJL = medial joint line; TAM = tibialis anterior muscle; BRM = brachioradialis muscle; TS = temporal summation; KOOS = Knee injury and Osteoarthritis Outcome Score; GLP-1 = glucagon-like peptide-1.

We found that inulin led to a higher proportion of individuals having improvements in proximal sensitisation (near the painful knee) compared to placebo, as measured by a higher PPT at the superolateral edge of the patella (SLP) at follow-up compared to baseline. In addition, inulin also improved TS, a measure indicative of increased spinal cord pain sensitivity and central sensitisation. Specifically, a higher proportion of individuals showed improvement in SLP in both inulin groups and their combination compared to placebo, while TS improvements were observed in the inulin only group and the combined inulin groups. In contrast, no such effects were seen in the PSE group (Figure 3a).

### 3.3. Associations of SCFA and GLP-1 Changes with Primary and Secondary Outcomes

Following on from the identified improvements in pain and pain sensitisation indices with inulin supplementation, we examined its effects on SCFAs as potential mediators of this relationship. We found that butyric acid levels increased from baseline to follow-up in the combined inulin groups compared to the non-inulin groups, whereas acetic acid levels remained unchanged across all groups. Propionic acid was excluded from the analysis because its values fell below the detection limit for most individuals. Interestingly, we also found that GLP-1 increased from baseline to follow-up in the inulin groups alone (Figure 3b).

To investigate whether changes in SCFAs may underlie the effects of inulin on pain and pain sensitisation, we examined correlations between SCFA levels and pain as well as functional traits in the inulin versus non-inulin groups. However, no significant correlations were identified, indicating that the effects of inulin are likely driven by a different mechanism (Figure 3c).

Given the lack of correlation between changes in SCFAs and changes in pain, we hypothesised that other molecular mediators might be involved. As recent evidence suggests that GLP-1 is regulated by fibre intake, and that it might have anti-nociceptive effects, we measured GLP-1 and assessed whether there was any correlation between changes in GLP-1 concentration in response to the interventions. These findings represent exploratory analyses conducted separately from the prespecified analyses. GLP-1 levels were positively associated with grip strength (Figure 3d) and inversely related to changes in physical performance KOOS function scores (Figure 3e), suggesting that improvements in muscle function may parallel GLP-1-linked metabolic responses in inulin arms.

## 4. Discussion

This 2 × 2 factorial RCT demonstrated that inulin, PSE, and their combination each had moderate to large effects in reducing pain compared to placebo, with both interventions exceeding the MCID threshold for NRS pain, suggesting clinically meaningful improvements. In addition, PSE effectively improved both squatting and walking ability, while inulin supplementation led to improvements in grip strength over time, as well as improved proximal and central sensitisation indices compared to placebo.

We confirmed the effectiveness of the digital PSE intervention in reducing pain and improving walking and squatting ability with moderate to large effects, consistent with our previous findings [20].

Inulin had a moderate effect on pain reduction in knee OA, consistent with the trend recently reported [19] in individuals with knee OA and comorbid obesity at 6 months. In fact, our effect size is comparable to observed effects in the Fortuna et al. study [19], despite our study showing these effects at just 6 weeks and involving a more gender-balanced cohort, compared to the 92% female participants in the Fortuna et al. study. In line with their findings, we also observed an improvement in grip strength at 6 weeks with inulin, reinforcing the idea of a gut–muscle connection [31], but we did not replicate the improvements in walking ability measured by the TUG test. This discrepancy may be due to ceiling effects, due to differences in the two populations, or possibly because improvements in walking ability may take longer to manifest.

We also found that inulin supplementation led to a higher proportion of individuals showing lower temporal summation and higher PPTs, which are indices of central and proximal sensitisation, compared to placebo. Previous research has linked gut microbiome alterations to pain in knee OA and fibromyalgia [32,33,34]. In animal models, restoring gut microflora reduced neuropathic-like and visceral pain, as well as pain sensitisation, independent of cognitive or anxiety-like behaviours [7,35]. Furthermore, in a mouse OA model, oligofructose restored beneficial microbes like Bifidobacterium, reduced joint inflammation, and lowered chondrocyte hypertrophy [36], processes involved in pain sensitisation. Similar benefits, including increased Bifidobacterium, were observed in rats given prebiotics and aerobic exercise [37]. Fortuna et al. [19] further found that higher Bifidobacterium levels correlated with improved grip strength and walking performance persisting at 3 months.

Although prebiotics influence the microbiome via SCFA production, we found no link between serum acetate or butyrate levels and pain or function, despite higher butyrate levels in the inulin group. This may reflect the limitations of using serum SCFA measurements, as they do not reflect levels in the gut [38]. Interestingly, inulin supplementation was associated with increased circulating GLP-1, which in turn correlated with improvements in grip strength and KOOS function, though these results should be considered exploratory. Further work is warranted to elucidate the mechanisms linking GLP-1 signalling to musculoskeletal outcomes and to determine whether targeting GLP-1 pathways could provide therapeutic benefit in knee OA.

We did not observe improvements in pain sensitisation indices with PSE alone, consistent with our previous findings [20]. This may be attributed to the possibility that improvements in pain sensitisation could predict responsiveness to PSE—specifically when comparing participants who show a decrease in NRS values following the intervention with those who show either no change or an increase—as previously reported [39]. However, exploring this hypothesis was beyond the scope of the present study.

Finally, we report significantly lower dropout rates for individuals with knee OA engaging with a purely nutritional intervention (3.6%) than for individuals engaging with a simple physiotherapy intervention (21%). Although the present study was not designed to formally compare acceptability or long-term adherence, these findings raise the hypothesis that nutritional interventions for OA might have higher acceptability and long-term adherence than physiotherapy-type interventions in some individuals. Together with the effects on grip strength and pain sensitivity, these findings support further investigation into the potential role of nutritional interventions for OA to reduce the burden of disease in the general population.

### 4.1. Generalisability

The demographic and basic clinical characteristics of participants in this study (e.g., age, sex, BMI, and pain) were similar to those in our previously reported RCT and aligned with individuals in population-based OA registers such as BOA, GLA:D, and JA, as well as patients eligible for total knee replacement (see generalisability section in [20]). However, we note that our results cannot be generalised to individuals with fibromyalgia or IBS, as they were excluded from the study.

### 4.2. Limitations

Despite being an RCT, substantially higher dropout rates in the PSE arms (21% vs. 3.6%) were identified during the study, necessitating targeted over-recruitment in these arms to maintain adequate sample size, resulting in modification of allocation probabilities across study arms. This sequential recruitment strategy resulted in some imbalance across study arms and potential temporal confounding, though baseline demographic and pain characteristics remained comparable across recruitment phases (Supplementary Table S6) and sensitivity analyses adjusting for time of recruitment showed consistent results (Supplementary Table S10). Our results demonstrate that inulin exerts a moderate effect on both pain reduction and pain sensitisation, an effect not previously reported in humans, as well as a moderate effect on grip strength. However, it is worth highlighting that the deviation from fixed allocation reduces the strength of causal inference relative to a fully balanced randomised design, and, although sensitivity analyses adjusting for recruitment phase yielded similar estimates, residual confounding related to sequential recruitment cannot be excluded.

Additionally, our measurement of GLP-1 was exploratory and not prespecified in the original protocol, undertaken after SCFA analyses proved insufficient to explain inulin’s effects. While these findings are intriguing and biologically plausible given recent evidence of GLP-1’s role in pain modulation and musculoskeletal health, they should be considered hypothesis-generating and require independent replication.

No synergistic effects between inulin and PSE were observed, yet it is important to note that a post hoc power analysis found that the study was underpowered to detect synergistic interactions (see Supplementary Materials). Therefore, no causal inference can be drawn from the absence of synergistic effects observed in the present study.

A key limitation is that participants could not be blinded to PSE allocation. Unlike pharmaceutical interventions, blinding to behavioural interventions such as exercise is inherently impossible with conscious participants who must actively engage in the treatment—a well-recognised challenge in physiotherapy trials [40]. This introduces potential expectancy effects, particularly for subjective outcomes like self-reported pain. However, this concern is partially mitigated by blinded outcome assessors, the inclusion of objective functional measures (30-CST, TUG, grip strength) and quantitative sensory testing (PPT, TS) that are less susceptible to bias, and the fact that inulin—to which participants were successfully blinded—produced comparable pain reductions, suggesting genuine physiological improvements beyond expectancy effects. Furthermore, the efficacy of exercise interventions for reducing knee OA pain is robustly established by extensive evidence, including a recent systematic review of 139 trials (12,468 participants) demonstrating that exercise consistently improves both pain and physical function across multiple comparison groups [41]. While the improvements observed in the PSE arms are consistent with the broader literature, expectancy effects and other sources of bias cannot be excluded. Another limitation is that we did not explicitly exclude participants with high fibre intake. However, this study was strengthened by the recording of dietary intake, allowing us to compare habitual fibre intake. Fibre intake, including from supplements, showed no significant differences between groups, indicating it likely had minimal impact on the outcomes. Additional limitations include the relatively short 6-week duration, which may be insufficient to capture the full effects of exercise typically observed in longer trials. Lastly, since there are no established MCIDs for PPT and TS, it is difficult to assess whether the improvements in pain sensitisation indices observed with inulin are clinically significant.

## 5. Conclusions

Our findings indicate that inulin supplementation was associated with moderate improvements in both pain and pain sensitisation indices, whereas digital PSE alone does not appear to influence pain sensitivity. In addition, inulin was associated with improvements in grip strength, paralleled by increases in GLP-1. These results provide a novel perspective on OA management, highlighting the potential for integrating dietary interventions alongside pharmacological treatments and promoting a more holistic approach to patient care.

## Figures and Tables

**Table 1:** Demographic, pain NRS and functional outcome characteristics of participants of the INSPIRE study at baseline by intervention allocation group.

Characteristic	Study Group				Statistical Difference Between Groups
**Demographics**	**Inulin**	**PSE**	**Inulin & PSE**	**Placebo**	
Sex (F%)	53.85%	65.00%	58.33%	51.85%	X2 (3 *df*) = 1.409 *p* = 0.703
n (F/M)	14/12	26/14	14/10	14/13	
Age (years) SD	66.54	67.75	66.71	69.59	F(3, 112) = 0.57 *p* = 0.64
SD	11.39	10.22	8.44	6.41	
BMI kg/m^2^	30.15	29.00	29.50	29.60	F(3, 112) = 0.065 *p* = 0.98
SD	3.75	7.15	3.52	4.65	
IMD ^1^ decile	6.65	6.75	7.00	7.04	F(3, 112) = 0.116 *p* = 0.95
SD	3.00	2.87	2.75	2.66	
**Functional** **Assessments**	**Inulin**	**PSE**	**Inulin** **& PSE**	**Placebo**	
Timed-up-and-go (TUG) (secs)	10.39	10.38	9.55	9.51	F(3, 112) = 0.478 *p* = 0.70
SD	4.03	5.05	2.01	2.19	
30-s sit-to-stand (30-CST)	10.60	11.30	11.58	10.88	F(3, 111) = 0.388 *p* = 0.76
SD	3.51	3.55	3.59	3.23	
Grip strength (kg)	37.97	33.77	35.90	35.29	F(3, 113) = 0.674 *p* = 0.57
SD	15.66	9.47	9.93	11.49	
**Pain**	**Inulin**	**PSE**	**Inulin** **& PSE**	**Placebo**	
Numerical Rating Scale (NRS)	4.06	4.21	3.92	3.52	F(3, 112) = 0.37 *p* = 0.77
SD	2.85	2.88	1.75	2.77	

^1^ Abbreviations: IMD = Index of Multiple Deprivation.

## Data Availability

The data presented in this study are openly available in [ClinicalTrials.gov] at [https://clinicaltrials.gov/study/NCT05670314?term=NCT05670314&rank=1&tab=results (accessed on 18 February 2026)], reference number [21].

## Supplementary Materials

The following supporting information can be downloaded at https://www.mdpi.com/article/10.3390/nu18050714/s1: Table S1: Inclusion and exclusion criteria; Table S2: Results of the ITT analyses using multiple imputation (m = 40) for missing week-6 data for primary and secondary outcomes; Figure S1: Convergence plots. The plots show the mean (left) and standard deviation (right) of the imputed values for each of the imputed variables (m = 40); Figure S2: Density plots. Red lines depict imputed data while the blue line is the distribution of the original data (m = 40); Table S3: Pain sensitisation indices (pressure pain thresholds and temporal summation) and KOOS function characteristics of participants of the INSPIRE study at baseline by intervention allocation group; Table S4: Comparison of main baseline characteristics of participants (age, sex, BMI and NRS pain) for those that completed the study compared to those that withdrew or were excluded from per-protocol analysis; Table S5: Recruitment timeline per arm. The number of participants recruited between months 1–6, months 7–12 and month 13 and beyond for each arm is shown; Table S6: Comparison of main baseline characteristics of participants (age, sex, BMI and NRS pain) by time of recruitment; Table S7: Raw absolute mean changes (±SD) in NRS pain score for each treatment arm; Table S8: Response rate by treatment arm (defining response as an improvement of NRS 2 or higher) and comparison to the placebo arm; Table S9: Change in quantitative sensory traits in each individual arm and comparison to the placebo arm; Table S10: Results of sensitivity analyses adjusting for time of recruitment for primary and secondary outcomes.

## Abbreviations

The following abbreviations are used in this manuscript:

OA	osteoarthritis
SCFAs	short-chain fatty acids
GLP-1	glucagon-like peptide-1
PSE	physiotherapy-supported exercise
Δ	baseline-adjusted between-group mean difference
RCT	randomised controlled trial
REC	Research Ethics Committee
CONSORT	Consolidated Standards of Reporting Trials
BMI	body mass index
JA	Joint Academy
NRS	Numerical Rating Scale
QST	quantitative sensory testing
PPT	pressure pain detection threshold
TS	temporal summation
30-CST	30 s sit-to-stand test
TUG	timed up and go
SDs	standard deviations
IMD	Index of Multiple Deprivation
CI	confidence interval
MCID	minimum clinically important difference
SLP	superolateral edge of the patella
KOOS	Knee injury and Osteoarthritis Outcome Score
ITT	intention-to-treat
