# Supplementary material for: Effect of Prebiotic Supplementation With and Without Physiotherapy on Pain and Pain Sensitivity in People with Knee Osteoarthritis
Source: Nutrients. 2026 Feb 24;18(5):714. doi: 10.3390/nu18050714 (PMC12986947; doi:10.3390/nu18050714)
Supplement: Supplementary file 1 [file nutrients-18-00714-s001.zip › nutrients-4135847-supplementary.pdf]

Supplementary methods:

Supplementary Table S1. Inclusion and exclusion criteria

Category	Criteria
**Inclusion criteria**	• Reported pain in or around a knee on most days for more than 3 months at the first visit.<br>• Self-reported diagnosis of knee osteoarthritis (OA).<br>• Aged > 18 years.<br>• Body mass index (BMI) between 18.5 and 39.9 kg/m².<br>• Willing and able to provide informed consent for participation.
**Exclusion criteria**	• Presence of psychosocial or gastrointestinal disorders (e.g., irritable bowel syndrome, inflammatory bowel disease, coeliac disease).<br>• Current use of immunosuppressants, anticoagulants, amiodarone, and/or perhexiline.<br>• Following or planning to start a specialised or commercially available weight-loss diet or programme.<br>• Pregnant or breastfeeding.<br>• History or current psychiatric illness (including clinical depression).<br>• Diagnosis of a neurological condition (e.g., epilepsy).<br>• Previous total knee replacement.<br>• Severe hip OA (on a waiting list for total hip arthroplasty).<br>• Diagnosis of inflammatory arthropathy or non-OA causes of knee pain (e.g., rheumatoid arthritis).<br>• Diagnosis of neuropathy or diabetes mellitus.<br>• Participation in another research study involving invasive procedures or inconvenience allowance within the past 3 months.

PPT and TS: Proximal sites tested with PPT were the medial joint line (3cm medially from medial edge of patella) and quadriceps (2cm above superolateral and superomedial edges of patella). Distant sites included tibialis anterior (5 cm distal and 1 cm lateral to the tibial tuberosity) and brachioradialis (5 cm medial and distal to the lateral epicondyle) on the arm opposite the painful knee. At the point when the pressure became painful, participants pressed a button and PPT was recorded. TS was assessed using a retractable blunt needle (256 mN Pinprick; MRC-Systems, Heidelberg, Germany). A single stimulus was applied to the rectus femoris, positioned 5 cm above the midpoint of the patella of the most painful knee, followed by 10 repetitive stimuli delivered at a frequency of 1 s^{-1} (one stimulus per second). Participants rated pain/sharpness intensity on a 0–10 Visual Analogue Scale after the single stimulus and after the average pain/sharpness rating across the repeated stimuli. Each test was performed twice, with a 2-minute break between repetitions and the average was taken.

Pain criteria and dropouts: Participants were provisionally allocated to groups following a telephone screening confirming they met the study's inclusion and exclusion criteria. Formal randomisation occurred only at the baseline visit, where eligibility was re-confirmed in person, including confirmation of the knee osteoarthritis (OA) pain criterion (pain on most days of the month for more than 3 months). Individuals who did not attend the baseline visit (n = 38) were not randomised and provided no data.

Among the 136 participants who were randomised, 117 completed both baseline and follow-up assessments, while 19 withdrew after baseline but before follow-up. Dropout rates were higher in the physiotherapy arms (17/81; 21%) than in the non-physiotherapy arms (2/55; 3.6%), representing a statistically significant difference ($\chi^2(1) = 7.2$, $p = 0.008$; Fisher's exact test $p = 0.008$). Recruitment was subsequently extended for the physiotherapy arm to achieve the target sample size.

Habitual diet and Pain medications: Habitual dietary patterns were recorded using four-day food diaries both before and after the intervention, with data extracted via the Nutritics platform. Comparisons of macronutrient and fibre intakes between the pre- and post-intervention revealed no significant differences across the four groups.

Medications use was recorded using medication records and questionnaires. There were no significant differences across the four groups in the number of pain medications received (opioids, NSAIDs, and antineuropathic).

Post hoc power analysis for synergistic effect: A post hoc power analysis using the *pwr.f2.test* function in R was conducted for the interaction of inulin and PSE in a multiple linear regression model with change in pain as the outcome. The analysis compared models with and without the interaction effect. The estimated effect size (Cohen's f^2) for the interaction was 0.02, corresponding to a small effect. Given the residual degrees of freedom (df =113) and $\alpha = 0.05$, the calculated power was 0.33, indicating that the study was underpowered to detect a synergistic interaction.

Handling of missing data and sensitivity analysis: A sensitivity analysis was conducted on an intention-to-treat (ITT) basis including all 136 randomised participants to assess the robustness of the complete-case (per-protocol) analysis. Of these, 19 participants had baseline-only data available. Missing week-6 outcome data for participants who withdrew early were imputed using multiple imputation by chained equations (MICE) with 40 imputations under a missing-at-random (MAR) assumption, employing predictive mean matching. Baseline pain or function scores were included as covariates to account for variation in initial severity.

Results from the ITT analysis with multiple imputation (**Supplementary Table 2**) were consistent with the per-protocol findings, confirming the robustness of observed treatment effects. Convergence of the chained equations was confirmed by inspection of trace plots for all variables, which showed stable means and standard deviations across iterations (**Supplementary Figure S1**). Density plots indicated that imputed distributions closely matched those of observed data (**Supplementary Figure S2**).

Supplementary Table S2. Results of the ITT analyses using multiple imputation (m = 40) for missing week-6 data for primary and secondary outcomes.

Outcome	Comparison	Estimate	SE	95% CI (lower to upper)	P-value
**PAIN (NUMERICAL RATING SCORE)**	*Inulin vs Placebo*	-1.14	0.55	-2.21 to -0.07	***0.039***
	PSE vs Placebo	-1.59	0.50	-2.56 to -0.62	***0.0017***
	Inulin & PSE vs Placebo	-1.64	0.55	-2.72 to -0.56	***0.0036***
**30 SEC CHAIR STANDS (30CST)**	*Inulin vs Placebo*	-0.15	0.87	-1.85 to 1.56	0.867
	PSE vs Placebo	2.62	0.79	1.07 to 4.16	***0.0012***
	Inulin & PSE vs Placebo	2.32	0.89	0.57 to 4.06	***0.0106***
**TIMED UP AND GO (TUG)**	*Inulin vs Placebo*	-0.32	0.37	-1.05 to 0.41	0.391
	PSE vs Placebo	-0.62	0.35	-1.29 to 0.06	0.0766
	Inulin & PSE vs Placebo	-0.31	0.38	-1.06 to 0.44	0.424
**GRIP STRENGTH**	*Inulin vs Placebo*	4.28	1.57	1.20 to 7.36	***0.0073***
	PSE vs Placebo	1.67	1.48	-1.22 to 4.56	0.260
	Inulin & PSE vs Placebo	2.09	1.61	-1.06 to 5.25	0.196

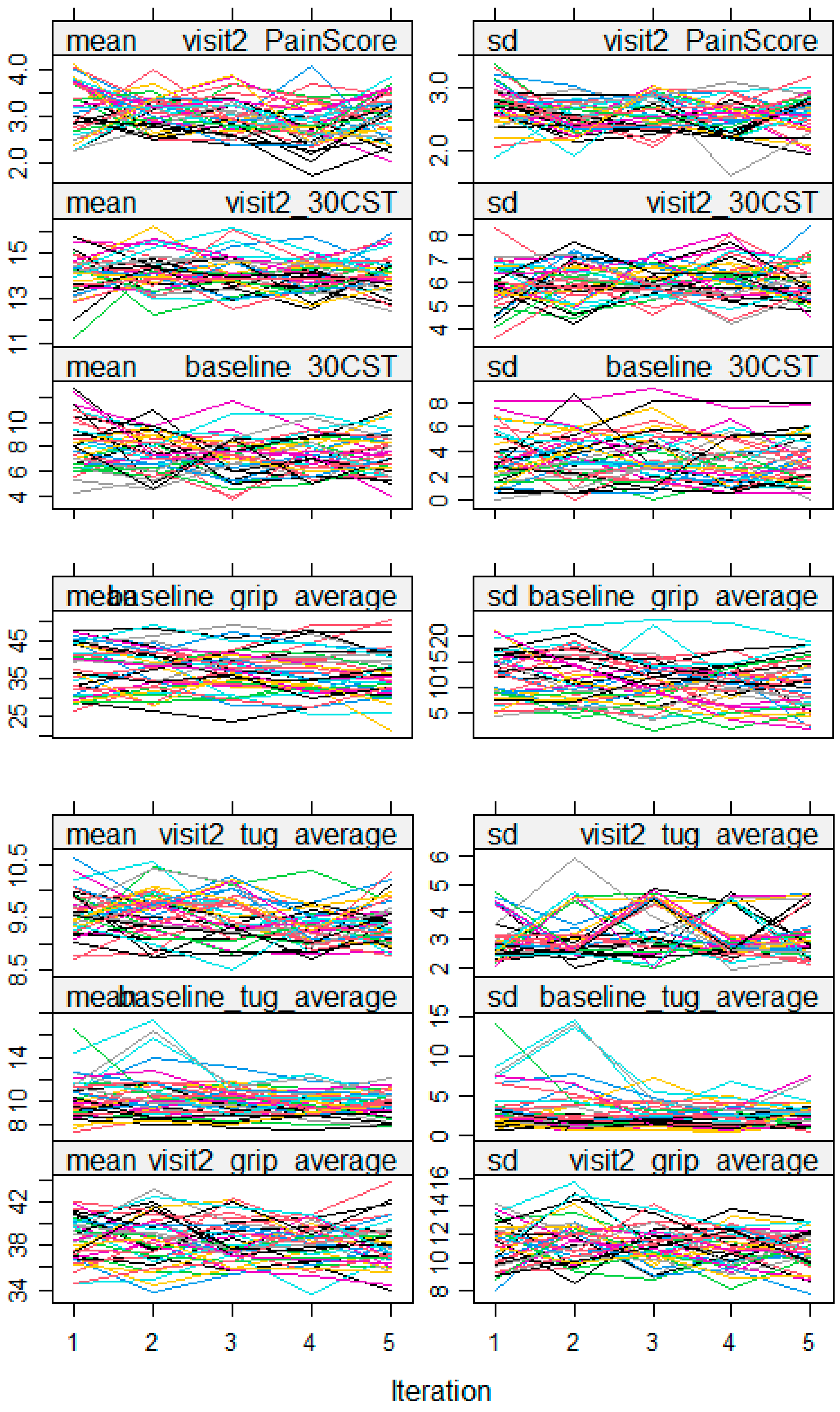

Supplementary Figure S1. *Convergence plots. The plots show the mean (left) and standard deviation (right) of the imputed values for each of the imputed variables (m=40).*

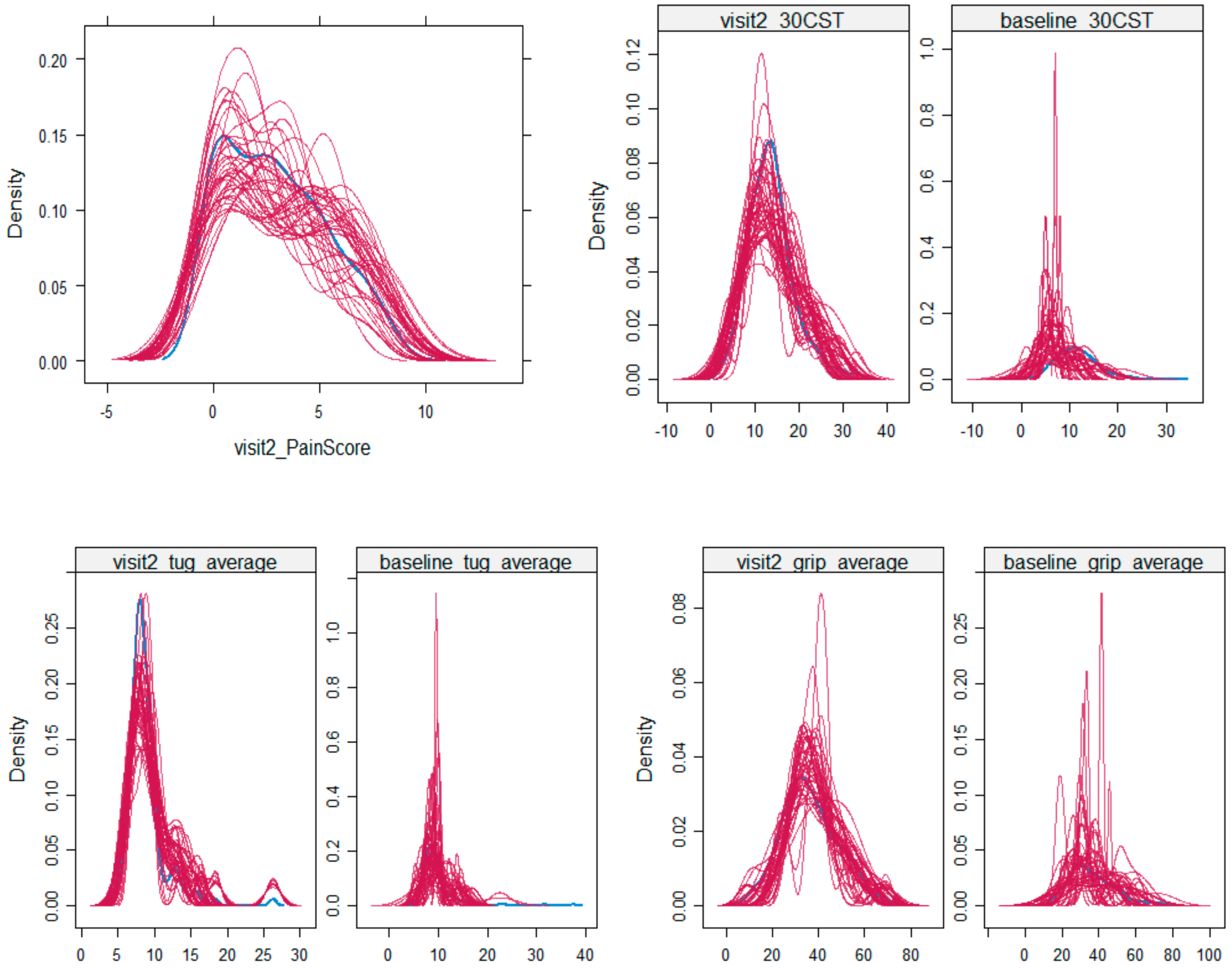

Supplementary Figure S2. *Density plots. Red lines depict imputed data while the blue line is the distribution of the original data (m=40)*

Supplementary Table S3. Pain sensitisation indices (pressure pain thresholds and temporal summation) and KOOS function characteristics of participants of the INSPIRE study at baseline by intervention allocation group

Characteristic		Study group				statistical difference between groups
**Proximal pressure pain thresholds (kPa)**						
		**Inulin**	**PSE**	**Inulin & PSE**	**Placebo**	
	superolateral patella	420.27	392.72	421.96	402.72	F(3,113)=0.141 P=0.93
	SD	241.35	177.09	198.26	215.36	
	superomedial patella	383.43	345.62	377.87	365.37	F(3,113)=0.244 P=0.865
	SD	268.03	139.04	166.01	191.70	
	medial joint line	389.20	351.48	392.95	375.26	F(3,113)=0.215 P=0.885
	SD	231.52	188.77	237.16	267.66	
**Distant pressure pain thresholds (kPa) & pain sensitivity index**						
		**Inulin**	**PSE**	**Inulin & PSE**	**Placebo**	
	brachioradialis muscle	342.93	285.11	299.83	286.26	F(3,113)=0.141 P=0.93
	SD	208.56	115.77	128.13	147.47	
	tibialis anterior muscle	396.22	313.47	387.30	391.93	F(3,113)=1.211 P=0.309
	SD	276.64	146.28	193.81	221.40	
	Temporal summation	1.53	1.63	1.91	1.49	F(3,113)=0.424 P=0.736
	SD	1.28	1.21	1.58	1.63	
**Knee injury and Osteoarthritis Outcome Score (KOOS)**						
		**Inulin**	**PSE**	**Inulin & PSE**	**Placebo**	
	**KOOS Function**	36.07	34.83	35.54	35.27	*F*(3,113) = 0.014 *p* = 0.99
	SD	25.67	25.50	25.42	25.03	

Supplementary Table S4. Comparison of main baseline characteristics of participants (age, sex, BMI and NRS pain) for those that completed the study compared to those that withdrew or were excluded from per-protocol analysis

Characteristic	Participants		statistical difference between groups
**Demographics and Pain**			
	**Completers**	**Non-completers**	
Sex (F%)	58.12%	53.49%	
n (F/M)	68/49	23/20	P=0.731
age (years) (SD)	67.69	67.20	
	9.48	9.60	P=0.363
BMI kg/m^2	29.48	29.44	
SD	5.32	6.67	P=0.489
Numerical Rating Score (NRS) Pain	3.96	3.29	P=0.499
SD	2.67	3.21	

Supplementary Table S5. Recruitment timeline per arm. The number of participants recruited between months 1-6, months 7-12 and month 13 and beyond for each arm is shown.

	A inulin	B PSE	C PSE+inulin	D placebo	total per stage
**MONTHS 1-6**	7	7	10	6	30
**MONTHS 7-12**	17	6	4	14	41
**MONTHS 13 +**	2	27	10	7	46
**TOTAL PER ARM**	26	40	24	27	117

Supplementary Table S6. Comparison of main baseline characteristics of participants (age, sex, BMI and NRS pain) by time of recruitment

CHARACTERISTIC		TIME OF RECRUITMENT			
**DEMOGRAPHICS AND PAIN**					statistical difference between groups (ANCOVA or χ^2)
		**mo 1-6**	**mo 7-12**	**mo 13+**	
	N	**30**	**41**	**46**	
	Sex (F%)	60.00%	58.54%	56.52%	
	n (F/M)	18/30	24/41	26/46	χ^2 = 0.095, df = 2, p = 0.954
	age (years)	69.00	67.00	67.46	F(2, 114) = 0.401, p = 0.671
	SD	7.62	10.71	9.63	
	BMI kg/m^2	28.70	30.27	29.28	F(2, 114) = 0.797, p = 0.453
	SD	4.28	4.20	6.72	
	Numerical Rating Score (NRS) Pain	3.83	3.85	4.13	F(2, 114) = 0.160, p = 0.852
	SD	1.91	2.94	2.90	

Supplementary Table S7. Raw absolute mean changes (±SD) in NRS pain score for each treatment arm

GROUP	INULIN	PSE	INULIN & PSE	PLACEBO
NO. OF PARTICIPANTS	26	40	24	27
MEAN (SD)	-0.73(2.32)	-1.25(2.69)	-1.21(1.76)	0.67(2.49)

Supplementary Table S8. Response rate by treatment arm (defining response as an improvement of NRS 2 or higher) and comparison to the placebo arm.

ARM	RESPONSE RATE	SAMPLE SIZE	95% CONFIDENCE INTERVAL	P-VALUE
**A- INULIN**	34.6%	26	(19.4%, 53.8%)	***0.041***
**B- PSE**	32.5%	40	(20.1%, 48.0%)	***0.044***
**C- PSE + INULIN**	37.5%	24	(21.2%, 57.3%)	***0.027***
**D - PLACEBO**	11.1%	27	(3.9%, 28.1%)	ref

Supplementary Table S9. Change in quantitative sensory traits in each individual arm and comparison to the placebo arm. The changes in pressure pain thresholds (PPTs) were compared between each arm for proximal (SLP, SMP, MJL) and distal (TAM and BRM) pressure pain thresholds and temporal summation scores. Mann Whitney test was used for comparisons, the U test and corresponding exact p-values are shown. In addition, the proportion of individuals who improved (had PPTs higher than at baseline or TS values lower than at baseline) was computed. The chi-squared test comparing these proportions for each trait and arm to the corresponding trait in placebo were computed.

Study Arm		Delta SLP	stat test vs D arm	Delta SMP	stat test vs D arm	delta MJL	stat test vs D arm	Delta TAM	stat test vs D arm	Delta BRM	stat test vs D arm	Delta TS	stat test vs D arm
Inulin	average	-29.89		-25.33		-31.72		-17.57		-69.33		-0.27	
	median	-29.90	U=292 P=0.301	10.80	U=314 P=0.397	-55.30	U=319.5 P=0.447	-3.40	U=300.5 P=0.276	-51.95	U=308.5 P=0.342	0.00	U=305.5 P=0.3084
	SD	178.82		125.00		135.13		164.41		121.40		1.44	
	Q1	-159.20		-78.90		-110.23		-116.65		-156.55		-1.00	
	Q3	111.23		52.18		50.23		80.15		10.58		0.00	
	% improved	42.3%	**chi2=6.73 P=0.009**	57.7%	chi2=0.94 P=0.332	38.5%	chi2=0.16 P=0.695	46.2%	chi2=0.02 P=0.900	30.8%	chi2=1.07 P=0.301	80.8%	**chi2=4.98 P=0.025**
PSE	average	-92.74		-41.25		-10.91		-7.74		-37.31		-0.22	
	median	-53.40	U=510.5 P=0.710	-41.60	U=553.5 P=0.938	-8.80	U=496 P=0.096	-16.60	U=456.5 P=0.1996	-37.25	U=548.5 P=0.884	0.00	U=486.5 P=0.3566
	SD	160.47		130.26		148.67		122.39		97.94		1.59	
	Q1	-141.63		-80.38		-80.13		-80.35		-83.78		-1.00	
	Q3	-53.40		-41.60		-8.80		-16.60		-37.25		0.00	
	% who improved	20.0%	chi2=0.78 P=0.377	35.0%	chi2=0.54 P=0.465	45.0%	chi2=0.81 P=0.369	45.0%	chi2=0.01 P=0.966	37.5%	chi2=0.29 P=0.593	70.0%	chi2=2.11 P=0.146
Inulin &PSE	average	-39.22		-22.28		-24.02		-20.66		-25.75		-0.77	
	median	-44.55	U=278 P=0.391	4.90	U=296.5 P=0.474	6.35	U=275 P=0.267	-22.05	U=292.5 P=0.4301	-24.45	U=319 P=0.760	-0.25	U=256.5 P=0.1423
	SD	245.44		133.92		163.94		184.61		153.55		2.29	
	Q1	-145.75		-79.15		-159.73		-82.60		-74.93		-2.00	
	Q3	69.80		53.95		74.95		54.90		59.85		1.00	
	% who improved	37.5%	**chi2=5.14 P=0.023**	54.2%	chi2=0.5 P=0.481	54.2%	chi2=2.33 P=0.127	45.8%	chi2=0.02 P=0.919	41.7%	chi2=0.05 P=0.838	66.7%	chi2=1.18 P=0.277
Placebo	average	-87.19		-46.73		-54.54		-47.36		-27.77		0.20	
	median	-61.80		-14.70		-50.90		-63.70		-12.70		0.00	
	SD	105.50		140.39		135.43		163.36		109.87		1.77	
	Q1	-128.85		-108.75		-139.65		-154.80		-76.85		-1.25	
	Q3	-25.5		41.65		28.95		57.80		41.65		1.50	
	% who improved	11.1%		44.4%		33.3%		44.4%		44.4%		51.9%	

Abbreviations: SLP = superolateral patella; SMP = superomedial patella; MJL medial joint line; TAM tibialis anterior muscle; BRM brachioradialis muscle; TS = temporal summation

Supplementary Table S10. Results of sensitivity analyses adjusting for time of recruitment for primary and secondary outcomes.

Outcome	Comparison	Estimate	SE	95% CI (lower to upper)	P-value
**PAIN (NUMERICAL RATING SCORE)**	*Inulin vs Placebo*	-1.11	0.55	-2.20 to -0.02	***0.046***
	PSE vs Placebo	-1.46	0.51	-2.48 to -0.45	***0.005***
	Inulin & PSE vs Placebo	-1.68	0.56	-2.79 to -0.57	***0.003***
**30 SEC CHAIR STANDS (30CST)**	*Inulin vs Placebo*	-0.02	0.84	-1.68 to 1.64	0.981
	PSE vs Placebo	2.85	0.77	1.31 to 4.39	***0.0004***
	Inulin & PSE vs Placebo	2.36	0.85	0.67 to 4.06	***0.007***
**TIMED UP AND GO (TUG)**	*Inulin vs Placebo*	-0.43	0.31	-1.05 to 0.18	0.165
	PSE vs Placebo	-0.62	0.29	-1.19 to -0.05	***0.034***
	Inulin & PSE vs Placebo	-0.34	0.31	-0.95 to 0.28	0.284
**GRIP STRENGTH**	*Inulin vs Placebo*	4.61	1.52	1.59 to 7.62	***0.003***
	PSE vs Placebo	1.19	1.42	-1.63 to 4.00	0.41
	Inulin & PSE vs Placebo	2.26	1.55	-0.81 to 5.33	0.15
